# Estrogen Receptor-Alpha (ESR1) Governs the Lower Female Reproductive Tract Vulnerability to *Candida albicans*

**DOI:** 10.3389/fimmu.2018.01033

**Published:** 2018-05-24

**Authors:** Laura Salinas-Muñoz, Raúl Campos-Fernández, Enrique Mercader, Irene Olivera-Valle, Carlota Fernández-Pacheco, Lara Matilla, Julio García-Bordas, Jennifer C. Brazil, Charles A. Parkos, Fernando Asensio, Maria A. Muñoz-Fernández, Andrés Hidalgo, Paloma Sánchez-Mateos, Rafael Samaniego, Miguel Relloso

**Affiliations:** ^1^Laboratorio de InmunoFisiología, Grupo Fisiopatología de la mujer, del embarazo, parto y puerperio, Instituto de Investigación Sanitaria Gregorio Marañón, Madrid, Spain; ^2^Servicio de Cirugía General, Sección Cirugía Endocrino-Metabólica, Hospital General Universitario Gregorio Marañón, Madrid, Spain; ^3^Animalario, Instituto de Investigación Sanitaria Gregorio Marañón, Madrid, Spain; ^4^Servicio de Anatomía Patológica, Hospital General Universitario Gregorio Marañón, Madrid, Spain; ^5^Department of Pathology, University of Michigan Medical School, Ann Arbor, MI, United States; ^6^Laboratorio InmunoBiología Molecular, Instituto de Investigación Sanitaria Gregorio Marañón, Madrid, Spain; ^7^Area of Cell and Developmental Biology, Fundación Centro Nacional de Investigaciones Cardiovasculares, Madrid, Spain; ^8^Laboratorio de Inmuno-oncología, Instituto de Investigación Sanitaria Gregorio Marañón, Madrid, Spain; ^9^Unidad de Microscopía Confocal, Instituto de Investigación Sanitaria Gregorio Marañón, Madrid, Spain

**Keywords:** estradiol, ESR1, progesterone, neutrophils, transepithelial migration, cervix

## Abstract

Estradiol-based therapies predispose women to vaginal infections. Moreover, it has long been known that neutrophils are absent from the vaginal lumen during the ovulatory phase (high estradiol). However, the mechanisms that regulate neutrophil influx to the vagina remain unknown. We investigated the neutrophil transepithelial migration (TEM) into the vaginal lumen. We revealed that estradiol reduces the CD44 and CD47 epithelial expression in the vaginal ectocervix and fornix, which retain neutrophils at the apical epithelium through the estradiol receptor-alpha. In contrast, luteal progesterone increases epithelial expression of CD44 and CD47 to promote neutrophil migration into the vaginal lumen and *Candida albicans* destruction. Distinctive to vaginal mucosa, neutrophil infiltration is contingent to sex hormones to prevent sperm from neutrophil attack; although it may compromise immunity during ovulation. Thus, sex hormones orchestrate tolerance and immunity in the vaginal lumen by regulating neutrophil TEM.

## Introduction

Neutrophils maintain mucosal immunity since they are very efficient at phagocytosing and killing invading microbes (1). However, neutrophil migration into the tissues must be carefully regulated because of the potential bystander tissue damage resulting from uncontrolled release of toxic metabolites (2). Neutrophils develop in the bone marrow (1) until they are mature to circulate in the blood (3). They are mobilized into the microcirculation by CXCR2-ligand gradients (4). Once extravasated, resident sentinel cells release proinflammatory mediators and chemoattractants that activate and guide neutrophils through the interstitium toward sites of infection/inflammation (5–7). In mucosal tissues, such as bladder, lung, and gut, epithelial cells produce chemoattractants that guide neutrophils through the interstitium until they reach the subepithelial space (8). Next, neutrophils cross the epithelial barrier in sequential steps: epithelial basement membrane crossing, initial adhesion to the epithelium, migration between epithelial cells, and detachment from the apical epithelial surface into the luminal space where they can combat invading microbes (9). Although neutrophil transepithelial migration (TEM) to the intestinal lumen has been somewhat characterized (10), how they migrate into the female reproductive tract (FRT) remains obscure (11).

In order to maintain a compromise between commensal and opportunistic microbiota, exogenous spermatozoa, and the threat of sexually transmitted pathogens, the vaginal mucosa is controlled by cyclic hormonal levels. Cervico-vaginal mucus and neutrophils protect epithelial cells from pathogens and create a microenvironment that fosters endogenous vaginal flora while deterring invasive microbial species that also target these regions, including Human Papiloma Viruses, *Candida albicans, T. vaginalis, C. trachomatis* and *N. gonorrhea* (12–14). Furthermore, sperm cells are pooled in the ectocervix and their survival must be facilitated if fertilization is to occur (15). To allow this, neutrophils disappear from the vaginal lumen during the ovulatory phase (16–19). However, these changes also create vulnerability to infection during ovulation (14). Although the leukocyte levels variation in the vaginal lumen during the ovarian cycle is well described in humans and mice (11, 17) and has important clinical implications (20), the mechanisms that regulate neutrophil influx to the vaginal lumen is an unsolved and long-standing question.

To study the role of hormones in regulating neutrophil migration in the vaginal lumen, we used a mouse model of candida infection, in the presence of estradiol (E2 follicular and ovulatory phase) or progesterone (P4 luteal phase) and analyzed the role of these hormones in each step of neutrophil migration in the FRT. We discovered that E2-treatment, through the estradiol receptor-alpha (ESR1), induces epithelial downregulation of CD47 and CD44, and retains neutrophils at the mucosa. We demonstrated how important molecules for neutrophil TEM in the gut (9) have a different regulation in the FRT to coordinate tolerance during ovulation to reconcile immunity and reproduction. Thus, the lack of neutrophils in the vaginal lumen during ovulation or following the use of E2-based therapies induces a vulnerability period that predisposes women to vaginal infections.

## Materials and Methods

### Ethical Approval

Procedures involving animals care complied with national and international laws and policies. The IiSGM Animal Care and Use Committee and Comunidad de Madrid approved all the procedures (PROEX 14714 and 27615). The Ethical Board of the IiSGM approved the human samples collection (CEIC-JV1.1/07-2014) and the consent obtained from the participants was both informed and written.

### *In Vivo* Hormonal Treatment, Vaginal Inoculation, Lavage, and Fungal Burden

Female BALB/c and C57BL/6 Wt and ESR1^−/−^ [B6N(Cg)-Esr1tm4.2Ksk/J JAX stock #026176]. ESR1^−/−^ mice are unfertile and uterus and vagina did not display cyclic changes (21). Mice were maintained under specific pathogen-free conditions in the Animal Facility of IiSGM. Four-week-old mice (13–15 g) were bilaterally ovariectomized under anesthesia (22) and given 2 weeks to recover. Females were randomly assigned to the groups and then injected subcutaneously with 0.02 mg of 17β-E2 or 0.2 mg of P4 (Calbiochem, Germany) dissolved in 100 µl of sesame oil (Sigma-Aldrich, USA) every 4 days. Hormone treatment was based on a previous report, and the dose was sufficient to maintain hormone concentrations in female mice (23). Seventy-two hours after first hormonal treatment, 2 × 10^6^ *C. albicans* blastoconidia in 20 µl of PBS were inoculated into the vagina. Vaginal secretions were collected by gently flushing the vaginal vault four times with 50 µl of sterile PBS using a 100 µl pipette tip inserted 5 mm in the vagina.

### *Candida albicans* 

The SC5314 *C. albicans* (ATCC MYA-2876) strain was grown on Sabouraud dextrose chloramphenicol agar plates (Conda, Spain) overnight at 30°C prior to the experiments.

### Vaginal Epithelial Cells *Ex Vivo* Analysis

Lower FRT was excised and dissociated using the Tumor Dissociation Kit (Miltenyi Biotec, Germany) following manufactures instructions. Samples were washed and the cells were stained (see below) for phenotypic analysis by flow cytometry. Epithelial cells were analyzed by gating on the Cdh1^+^ cells.

### Flow Cytometry

Cellular phenotypic analysis was carried out by direct immunofluorescence with the following antibodies: LY6G (eBioscience, USA), F4/80 (eBioscience, USA), CD11b (eBioscience, USA), CXCR4 (Santa Cruz Biotech, USA), metallomatrix proteinase 9 (MMP9) (R&D Systems, USA), CD44 (Miltenyi Biotec, Germany), Sirpα (Biolegend, USA), CD47 (Miltenyi Biotec, Germany), CDH1 (Biolegend, USA), and CD55 (Miltenyi Biotec, Germany). All incubations were done in the presence of 50 µg/ml mouse IgG, the same isotype control antibody was always included as a negative control, and dead cells were excluded by 7-amino-actinomycin D staining (Sigma, USA). Flow cytometry was performed with a Gallios device (Beckman Colter, USA) and data were analyzed using Flowjo software (Tree Star, Inc., USA). Cells were counted using Flow-Count fluorospheres (Beckman Coulter, USA) following the manufacturer’s instructions.

### Confocal Microscopy

Whole FRT tissues were embedded in Tissue-Tek OCT (Sakura, Netherlands). Eight-micrometer sections were fixed with acetone, blocked (50 µg/ml mouse IgG and 10% BSA) and stained with (1–5 µg/ml) the antibodies: LY6G (ebioscence, USA), MMP9 (R&D Systems, USA), CD44 (Miltenyi Biotec, Germany), Sirpα (Biolegend, USA), CD47 (Miltenyi Biotec, Germany), CDH1 (Biolegend, USA) and CD55 (Miltenyi Biotec, Germany). For FRT screening and *in vivo* protein expression quantification, tissues were usually triple-stained and imaged using the glycerol ACS APO 20× NA 0.60 immersion objective of a confocal fluorescence microscope (SPE, Leica Microsystems), maintaining the acquisition settings all over the process for each sample and among samples, as previously described (23, 24). Mean fluorescence intensities (MFI) were assessed at multiple regions of interest (10–15 by field) randomly depicted at specific areas of the FRT epithelium. Namely, the supraepithelial layer for CD44 and the middle layer of the stratified epithelium for CD47. For single-cell quantification, LY6G+ stained neutrophils were segmented and MFI of different markers quantified at matched cells. All quantifications, including line-profiles, were performed using the FIJI software (NIH).

### Human PMN, Mouse Neutrophils Isolation, and Depletion

PMNs were isolated from whole blood obtained from healthy donors by 3-layer Percoll gradient (Amersham Pharmacia Biotech, Sweden) (25). PMNs −97% pure and 99% viable (Figure S1 in Supplementary Material) were placed at 1 × 10^7^ cells/ml in complete RPMI 1640 (10% heat inactivated fetal calf serum, 1 mM sodium pyruvate, 1% amino acids, 2 mM l-glutamine, 50 µM 2-mercaptoethanol, and 15 mM HEPES). Mouse neutrophils were isolated from bone marrow cells or vaginal lavage (23) by anti-Ly-6G kit (MiltenyiBiotec, Germany) following manufactures instructions. Mouse neutrophil depletion was done by intravenously injection of 200 µg rat anti-mouse LY6G-1A8 or rat isotype control antibodies (Bio-X-Cell) in 0.1 ml sterile PBS (26). *C. albicans* vaginal inoculation was performed 24 h after the first antibody injection. Neutrophil depletion was confirmed by flow cytometry in the blood and vaginal lavage fluid (23).

### Transepithelial Migration

VK2/E6E7 (ATCC^®^CRL-2616TM) cells were grown on collagen-coated permeable 0.33-cm^2^ polycarbonate filters 5 µm pore size (Costar, USA). Confluent inverted VK2 monolayers were pretreated apically with 10 µg/ml of GM35 (27) or isotype control binding antibody (ebioscience, USA) for 20 min at room temperature. 1 × 10^6^ PMNs were added to the upper part of the transwell and allowed to migrate for 1 h at 37°C in the presence of 100 nM fMLF (Abcam, USA) in the physiologically relevant basolateral-to-apical direction (27, 28). Migrated PMNs were assessed at the lower chamber by flow cytometry.

### Statistical Analysis

The test used to determine significance between the treatments in each experiment can be found in the figure legends. GraphPad Prism 5 (GraphPad Software, Inc., USA) was used to determine statistical significance. A *p*-value <0.05 was considered significant.

## Results

### Estradiol Prevents Neutrophil Recruitment Into the Vaginal Lumen

**Figure 1:**
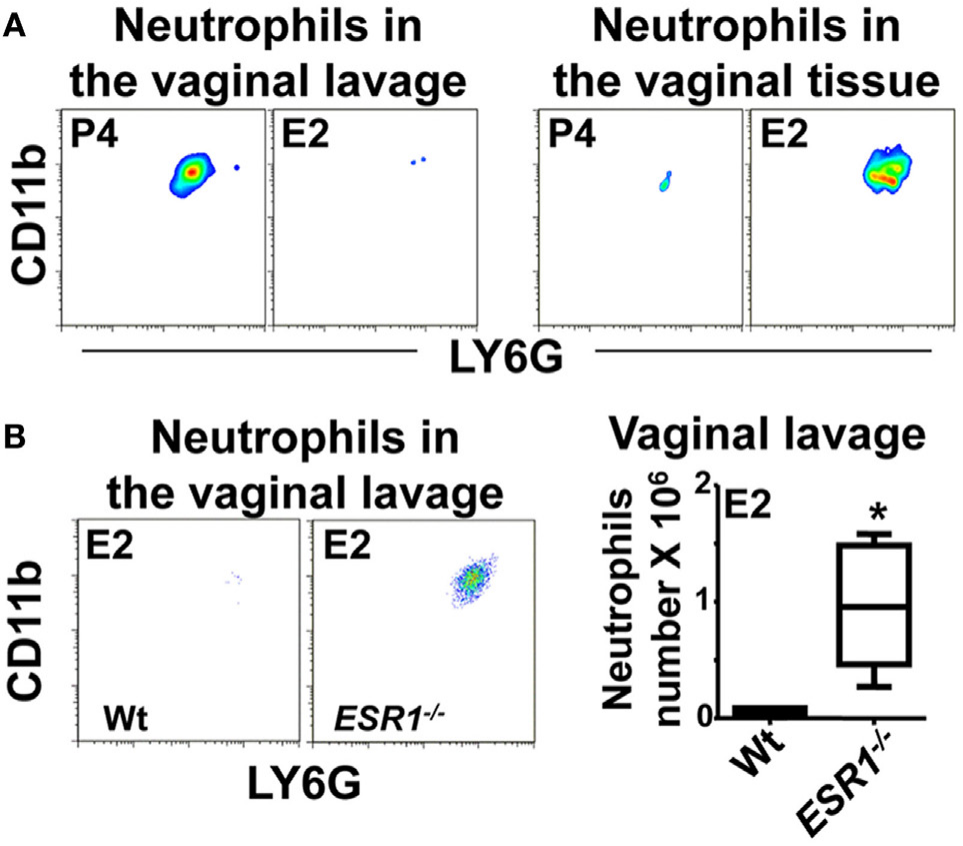
Neutrophils in the vaginal lavage. Hormone-treated ovariectomized mice were *Candida albicans* inoculated in the vagina for 6 h. **(A)** Representative flow cytometry plots of the neutrophils in the vaginal lavage and lower female reproductive tract tissue (*n* = 5). **(B)** Representative flow cytometry plots and quantification of the neutrophils in the vaginal lavage of estradiol treated *ESR1*^−^*^/^*^−^ and Wt mice challenged with *C. albicans*. Data are expressed as box and whiskers 10–90 percentile (*n* = 5). *p < 0.05 Mann–Whitney. Abbreviations: E2, estradiol; P4, progesterone.

To study the role of sex hormones in neutrophil influx into the vaginal lumen, we set up a model of *C. albicans* vaginal infection in hormone-treated mice. We previously detected a significant increase of neutrophils in the vaginal lavage of P4-treated mice (~25-fold) 6 h after infection compared with non-infected mice; in contrast, neutrophil infiltration was residual in E2-treated infected mice (Figure 1A) (23). Inhibition of neutrophil influx to the vaginal lumen in E2-treated mice could be due to a diminished recruitment from the vascular beds into the stromal layer or an impeded TEM. To discriminate between these possibilities, we excised the lower FRT after vaginal lavage, dissociated the tissue, and quantified neutrophils by flow cytometry. We detected neutrophils in the vaginal lavage but not in the tissue of P4-treated infected mice; however, E2-treated mice showed minimal infiltration in the vaginal lavage and considerable neutrophil accumulation in the lower FRT tissue (Figure 1A). Moreover, E2-treated *ESR1*^−/−^ mice showed higher neutrophil numbers (~15-fold) in the vaginal lavage than the Wt, indicating that ESR1 plays a key role in the downregulation of neutrophil migration across the vaginal epithelium (Figure 1B).

### Estradiol Induces Neutrophil Accumulation at the Fornix and Ectocervix

**Figure 2:**
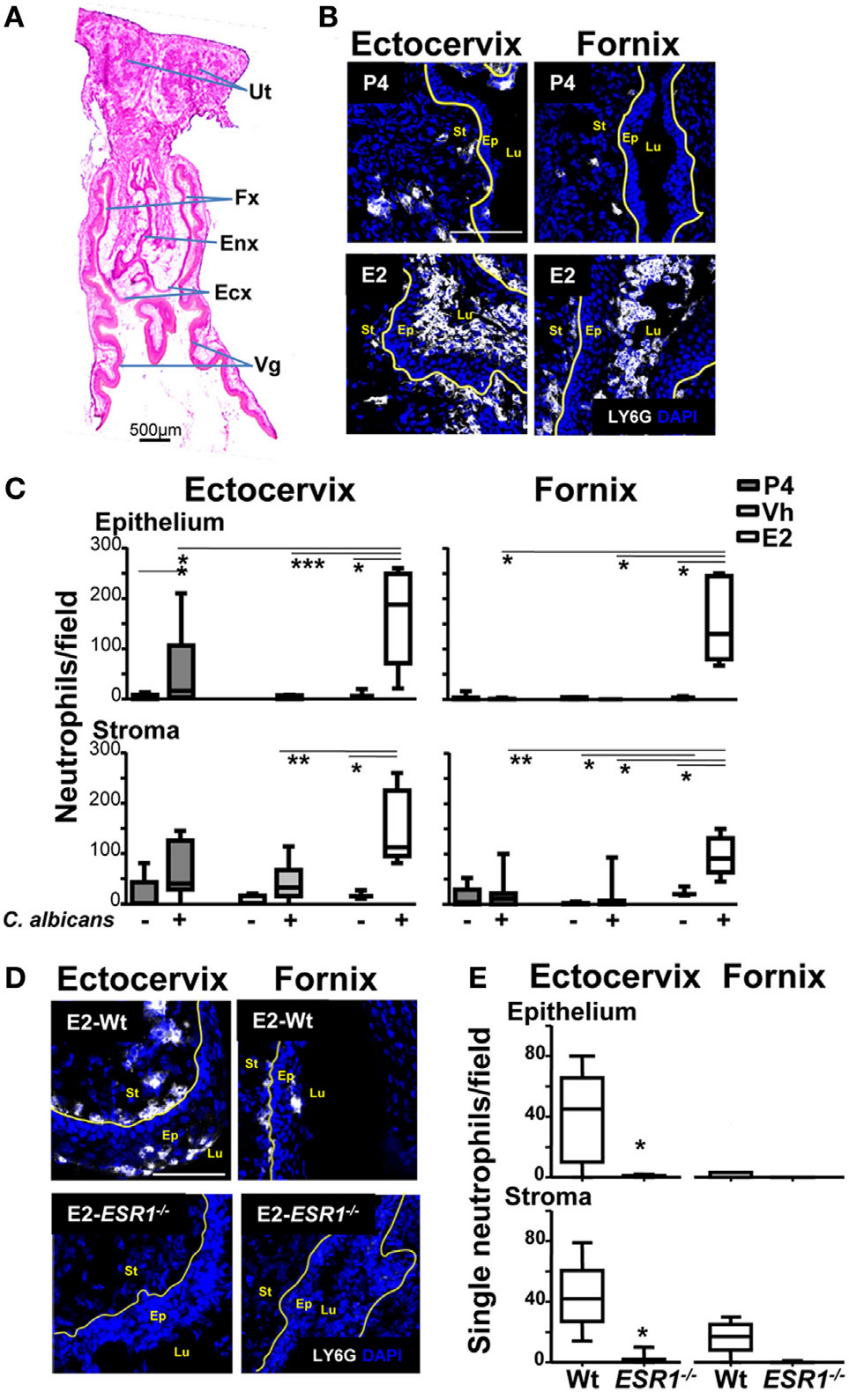
Neutrophils in the female reproductive tract (FRT). Hormone-treated ovariectomized mice were mock or *Candida albicans* inoculated in the vagina for 6 h. **(A)** Photomicrograph of the FRT. **(B)** FRT indicating anatomical regions with LY6G-stained neutrophils (white). **(C)** LY6G+ cells/field in the lower FRT epithelium and stroma. Data were calculated in at least three different sections of each sample (*n* = 5). **(D)** Neutrophils in the vaginal tissue of estradiol treated *ESR1*^−^*^/^*^−^ and Wt mice challenged with *C. albicans*. Representative photographs of ectocervix and fornix stained with LY6G (neutrophils white) and **(E)** quantification of the neutrophils. Data are expressed as box and whiskers 10–90 percentile (*n* = 5). *p < 0.05, **p < 0.01, and ***p < 0.001 Mann–Whitney. Yellow line indicates the lamina propia. Scale bar, 200 µm. Abbreviations: E2, estradiol; Vh, Vehicle; P4, progesterone; Ut, uterus; Fx, fornix; Enx, endocervix; Ecx, ectocervix; Vg, vagina; Lu, lumen; Ep, epithelium; St, stroma.

To determine at which area neutrophil migration was being blocked, we excised the lower FRT after vaginal lavage and quantified neutrophil density at the subepithelial stroma and at the epithelium along the FRT tissue by confocal microscopy. The lower FRT can be divided in two histological regions: (1) the introitus, whose epithelium resembles skin and, (2) the vagina, ectocervix, and fornix (the transition between the vagina and the ectocervix), which are covered by a stratified squamous epithelium. In contrast, the upper FRT (endocervix, uterus, and oviduct) consists of a simple columnar epithelium (Figure 2A) (29). We detected a higher density of neutrophils in E2-treated compared with vehicle (Vh) and P4-treated mice both in the fornix and ectocervix. In addition, neutrophil accumulation was observed in the stroma and attached to the apical surface of the epithelium following vaginal lavage. We detected less neutrophil accumulation in the vagina, endocervix, and uterus than in the fornix and ectocervix (Figures 2B,C; Figures S2A,B in Supplementary Material). Furthermore, E2-treated *ESR1*^−/−^ mice showed lower neutrophil numbers in the fornix (~6-fold) and ectocervix (~36-fold) epithelium than the Wt (Figures 2D,E), in accordance with the vaginal lavage in the Figure 1B. Thus, we demonstrate that E2 arrests neutrophils in the ectocervix and fornix, which may explain the absence of neutrophils in the vaginal lumen of E2-treated mice (Figure 1A). Therefore, we aimed to investigate how E2 regulates neutrophil migration across the vaginal epithelial barrier *in vivo*.

### Effect of Female Sex Hormones on Neutrophil Expression of Mediators of TEM

**Figure 3:**
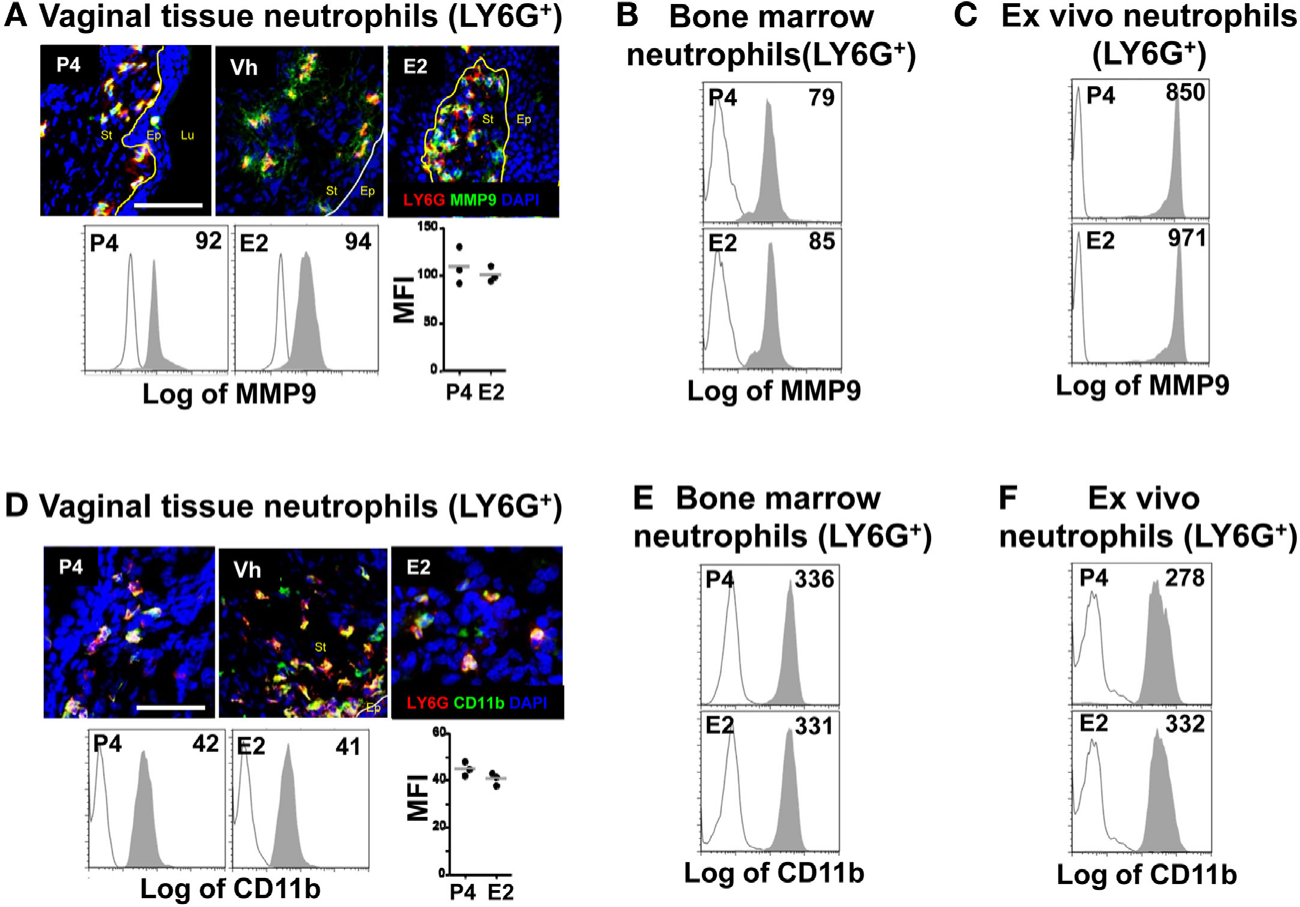
Hormone-treated ovariectomized mice were mock or *Candida albicans* inoculated in the vagina. **(A)** Representative pictures of sections assayed for metallomatrix proteinase 9 (MMP9) (green) and LY6G+ (red). Representative flow cytometry plots and quantification of the MMP9 expression in LY6G+ neutrophils from the lower female reproductive tract (FRT) of hormone-treated mice. MMP9 expression in LY6G+ neutrophils from *in vivo* hormone treated mice, **(B)** bone marrow, and **(C)** *ex vivo* treated neutrophils. **(D)** Representative pictures of sections assayed for CD11b (green) and LY6G+ (red). Representative flow cytometry plots and quantifications of the lower FRT neutrophils from hormone-treated mice. CD11b expression in LY6G+ neutrophils from *in vivo* hormone treated mice, **(E)** bone marrow, and **(F)** *ex vivo* treated neutrophils. Isotype control mAb (line) and indicated mAb (gray filled lines). Scale bar, 50 µm. Each circle represents a mouse in **(A,D)**, *n* = 4 in **(B,E)** and *n* = 5 in **(C,F)**. Abbreviations: E2, estradiol; Vh, vehicle; P4, progesterone; Lu, lumen; Ep, epithelium; St, stroma.

Neutrophil TEM is a multistep process; it starts with the initial crossing of the basement membrane and engagement of epithelial cells. Neutrophils secrete MMP9 to degrade collagen IV at the basement membrane (7). Next, the neutrophil integrin CD18/CD11b mediates the initial contact with epithelial cells, as shown in the lung, intestine, and bladder (9). Although E2-treatment resulted in accumulation of most neutrophils along the supra-epithelium, we detected accumulations in the subepithelium of the fornix and ectocervix. We, therefore, hypothesized that E2-treatment delays crossing of the basement membrane and/or engagement of the epithelial cells. We detected similar levels of MMP9 and CD11b in LY6G+ neutrophils of P4-, Vh, and E2-treated mice in the vagina, fornix, and ectocervix by confocal microscopy, as well as *in vivo* and *ex vivo* treatment by flow cytometry (Figures 3A–F). Suggesting that changes in these proteins are unrelated to the observed accumulation of neutrophils under the epithelium in the hormone-treated mice.

**Figure 4:**
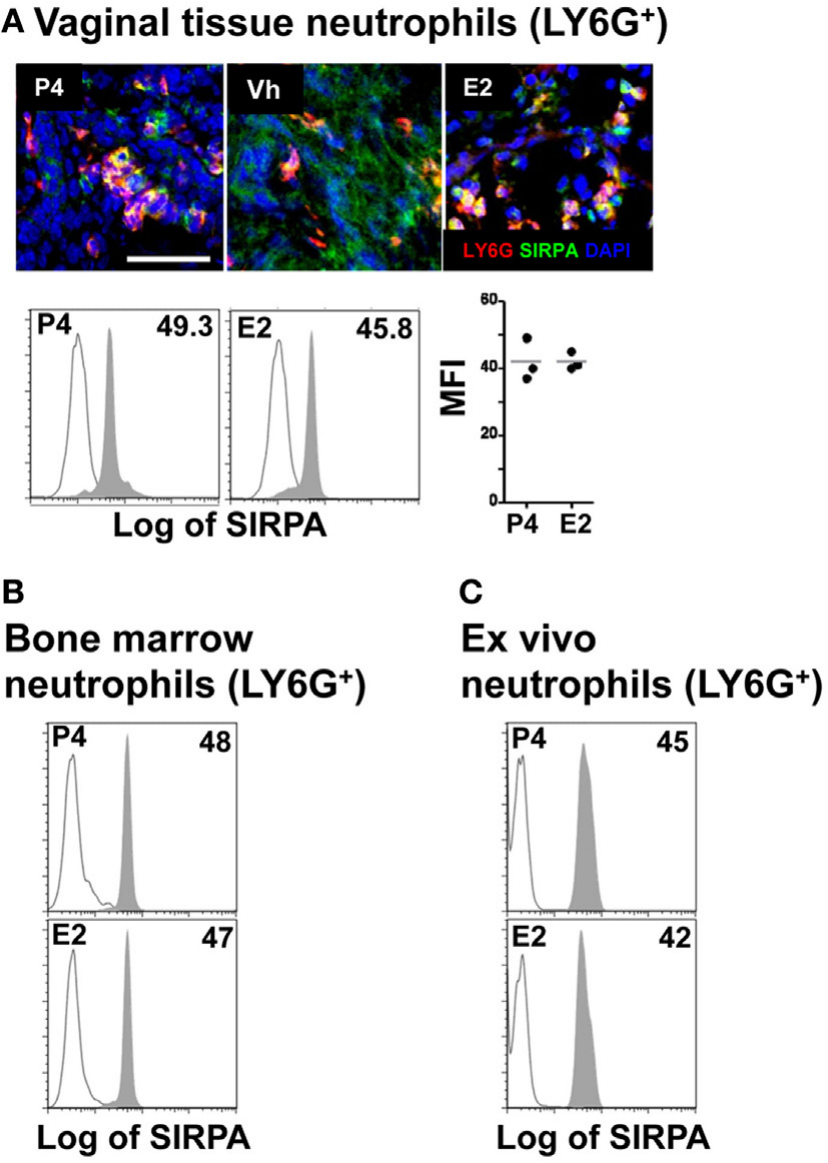
Hormone-treated ovariectomized mice were mock or *Candida albicans* inoculated in the vagina. **(A)** Representative pictures of sections assayed for SIRPA (green) and LY6G+ (red). Representative flow cytometry plots and quantification of the SIRPA expression in LY6G+ neutrophils from the lower female reproductive tract of hormone-treated mice. SIRPA expression in LY6G+ neutrophils from *in vivo* hormone-treated mice, **(B)** bone marrow, and **(C)** *ex vivo* treated neutrophils. Scale bar, 50 µm. Isotype control mAb (line) and indicated mAb (gray filled lines). Each circle represents a mouse **(A)**, *n* = 4 in **(B)**, and *n* = 5 in **(C)**. Abbreviations: E2, estradiol; Vh, vehicle; P4, progesterone.

After the initial contact of neutrophils with the epithelium, key protein–protein interactions facilitate movement through the paracellular space between adjacent epithelial cells. One of the epithelial receptors important for this process is CD47 (30). Disruption of the interaction between neutrophil SIRPA (signal-regulatory protein α or CD172a) and epithelial CD47 delays neutrophil TEM into the intestine (31). Analysis of SIRPA expression by vaginal neutrophils, however, revealed no differences in SIRPA expression in neutrophils in hormone-treated mice by confocal microscopy as well as *in vivo* and *ex vivo* treatment by flow cytometry (Figures 4A–C). This again suggests that neutrophil SIRPA is not subjected to regulation in E2-treated mice. In summary, our data suggest that E2 does not regulate the neutrophil TEM by acting on the neutrophils because E2-treatment did not affect neutrophil expression of the major receptors, CD11b, MMP9, and SIRPA.

**Figure 5:**
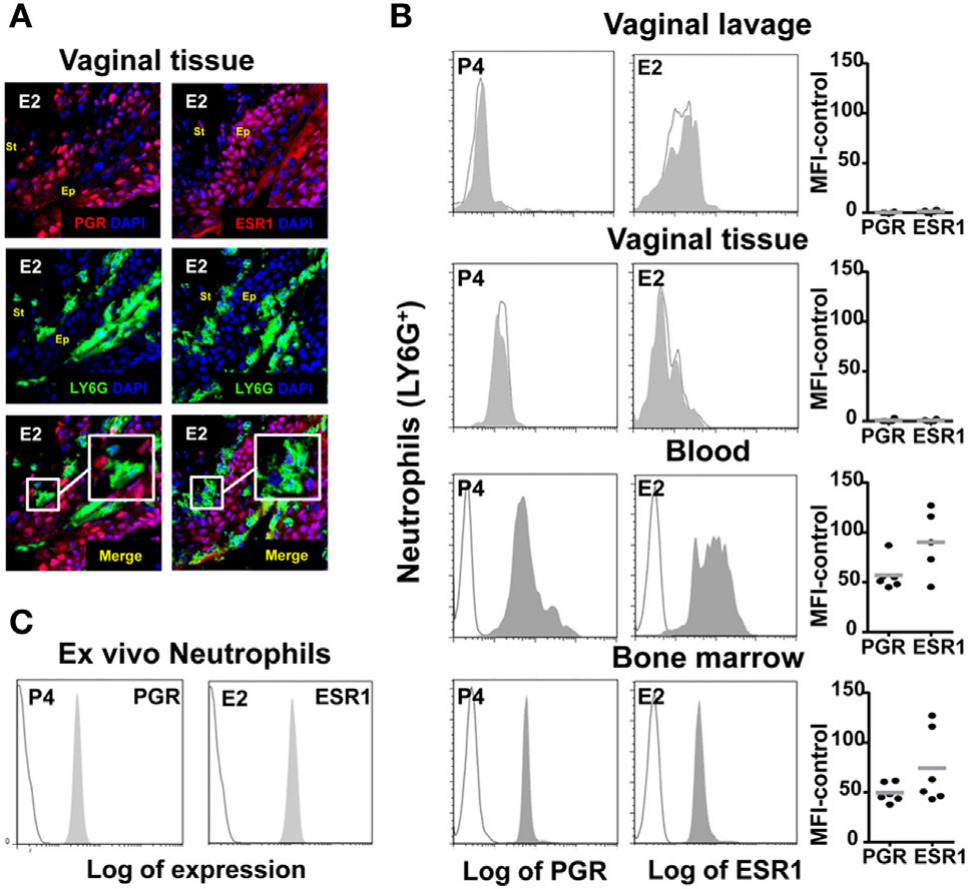
Hormone-treated ovariectomized mice were *Candida albicans* inoculated in the vagina. **(A)** Representative pictures of sections assayed for LY6G+ (green) and estradiol nuclear receptor ESR1 (red) or progesterone nuclear receptor PGR (red). **(B)** Representative flow cytometry plots neutrophils (LY6G+) assayed for ESR1 and PGR expression from vaginal lavage, lower female reproductive tract, blood, and bone marrow cells from hormone-treated mice. Each circle represents a mouse. **(C)** Bone marrow neutrophils were isolated and *ex vivo* hormone-treated for 12 h and assayed for ESR1 and PGR expression. A representative experiment of three independent tests is shown. Isotype control mAb (line) and indicated mAb (gray filled lines). Abbreviations: E2, estradiol; P4, progesterone; Ep, epithelium; St, stroma.

To better understand these findings, we looked for estradiol and progesterone nuclear receptor expression in neutrophils from the lower FRT. Although it has been previously reported that circulating neutrophils express estradiol and progesterone receptors (23, 32), we could not detect any protein receptor expression in neutrophils from the ectocervix and fornix, whole FTR, or vaginal lavage; in contrast, we detected expression of both nuclear receptors in the epithelial cells and neutrophils from blood, bone marrow, and *ex vivo* hormone treated (Figures 5A–C). This could explain why hormones unaffected neutrophils and suggested that sex hormones could regulate neutrophils by acting directly on the vaginal epithelial cells.

### Female Sex Hormones Act on the Epithelium to Regulate Neutrophil TEM

**Figure 6:**
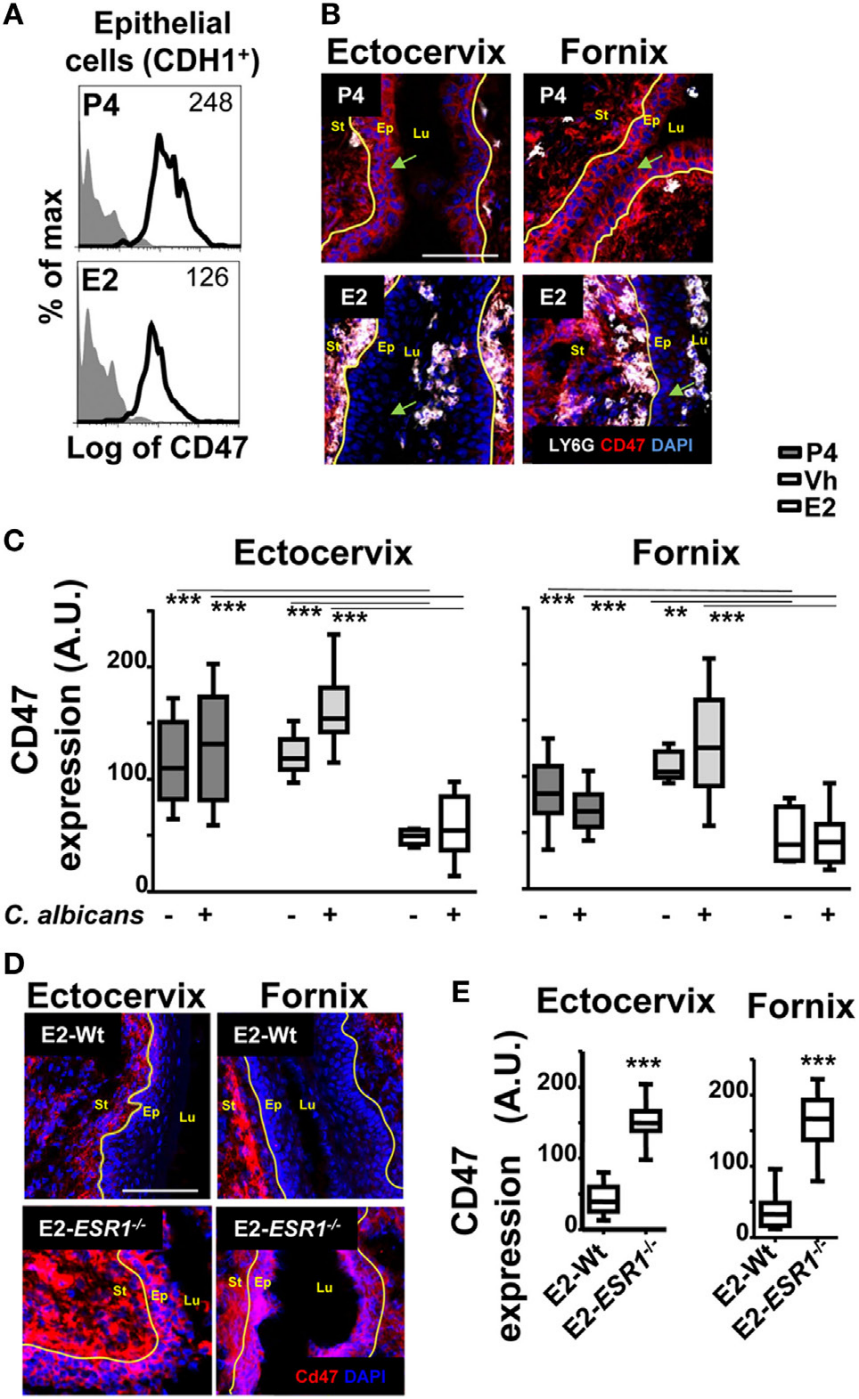
CD47 expression in the female reproductive tract (FRT). Hormone-treated ovariectomized mice were mock or *Candida albicans* inoculated in the vagina. **(A)** Representative flow cytometry plots of the lower FRT epithelial cells from hormone-treated mice (*n* = 3). **(B)** FRT with indicated anatomical regions stained for CD47 (red) or neutrophils (white). **(C)** CD47 expression in the basolateral epithelium. Data were calculated in at least three different points in three different sections of each sample (*n* = 5). **(D)** Estradiol treated *ESR1*^−^*^/^*^−^ and Wt mice challenged with *C. albicans*. Representative photographs of ectocervix and fornix stained with CD47 (red) and **(E)** quantification (*n* = 5). Data are expressed as box and whiskers 10–90 percentile. **p < 0.01 and ***p < 0.001 Mann–Whitney. Isotype control mAb (gray filled lines) and indicated mAb (lines). Yellow line indicates the lamina propia. Scale bar, 200 µm. Abbreviations: E2, estradiol; Vh, vehicle; P4, progesterone. Lu, lumen; Ep, epithelium; St, stroma; A.U., arbitrary units.

To study the role of sex hormones on epithelial cells and the regulation of the neutrophil passage through the lateral intercellular space between epithelial cells, we focused on CD47 expression (30, 33). We detected lower surface expression of CD47 in *ex vivo* FRT epithelial cells isolated from E2 compared to P4-treated mice, as assessed by flow cytometry (Figure 6A). To determine its exact anatomical distribution, we examined CD47 expression in epithelial cells of the lower FRT. We detected stronger expression of CD47 in P4- and Vh-treated compared with E2-treated epithelium in the vagina (~4-fold), ectocervix (~2-fold), and fornix (~2-fold). This differential expression was observed with and without fungal infection (Figures 6B,C; Figure S3A in Supplementary Material), indicating an E2-dependent and candida independent effect. Likewise, E2-teated *ESR1*^−/−^ mice expressed elevated levels of CD47 in the ectocervix and fornix; thus indicating that the effect was fully ESR1-dependent (Figures 6D,E). In conclusion, E2 downregulates CD47 expression along the ectocervix and fornix epithelial cells in E2-treated mouse, which coincides with E2-dependent subepithelial accumulation of neutrophils. Hence, lower levels of epithelial CD47 expression might contribute to the negative regulation of neutrophil migration into the vaginal lumen induced by E2, and their retention in the ectocervix and fornix epithelium.

### Estradiol Downregulates Epithelial Cell CD44 Expression

**Figure 7:**
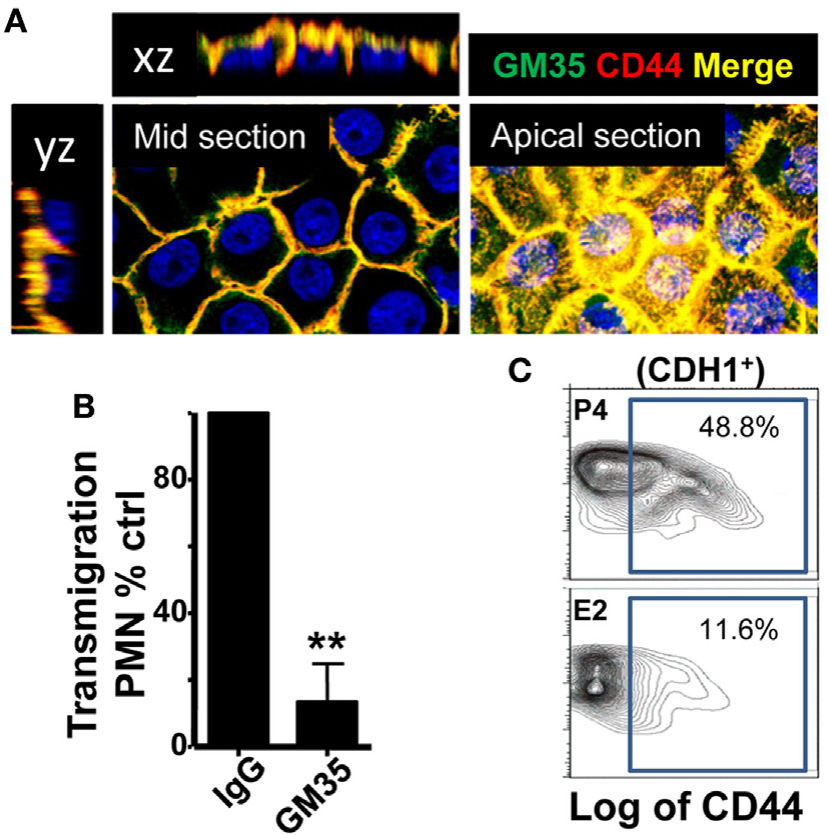
CD44 expression in the vaginal cells. **(A)** Confocal microscopy images of non-permeabilized VK2 monolayers co-stained with GM35 mAb (green) and CD44 (red). A representative picture of three independent tests is shown. **(B)** Percentage of control PMNs migrated in response to fMLF. Confluent VK2 monolayers were pre-treated apically with isotype control or GM35 mAb before PMNs were added to the basolateral surface. A representative experiment of 3 independent tests is shown. Data are expressed as mean ± SD. **p < 0.01 Unpaired t test with Welch’s correction. **(C)** Representative flow cytometry plots of the CD44 expression in the lower female reproductive tract epithelial cells from hormone-treated mice. Data are expressed as percentage of total cells in the gate (*n* = 3).

**Figure 8:**
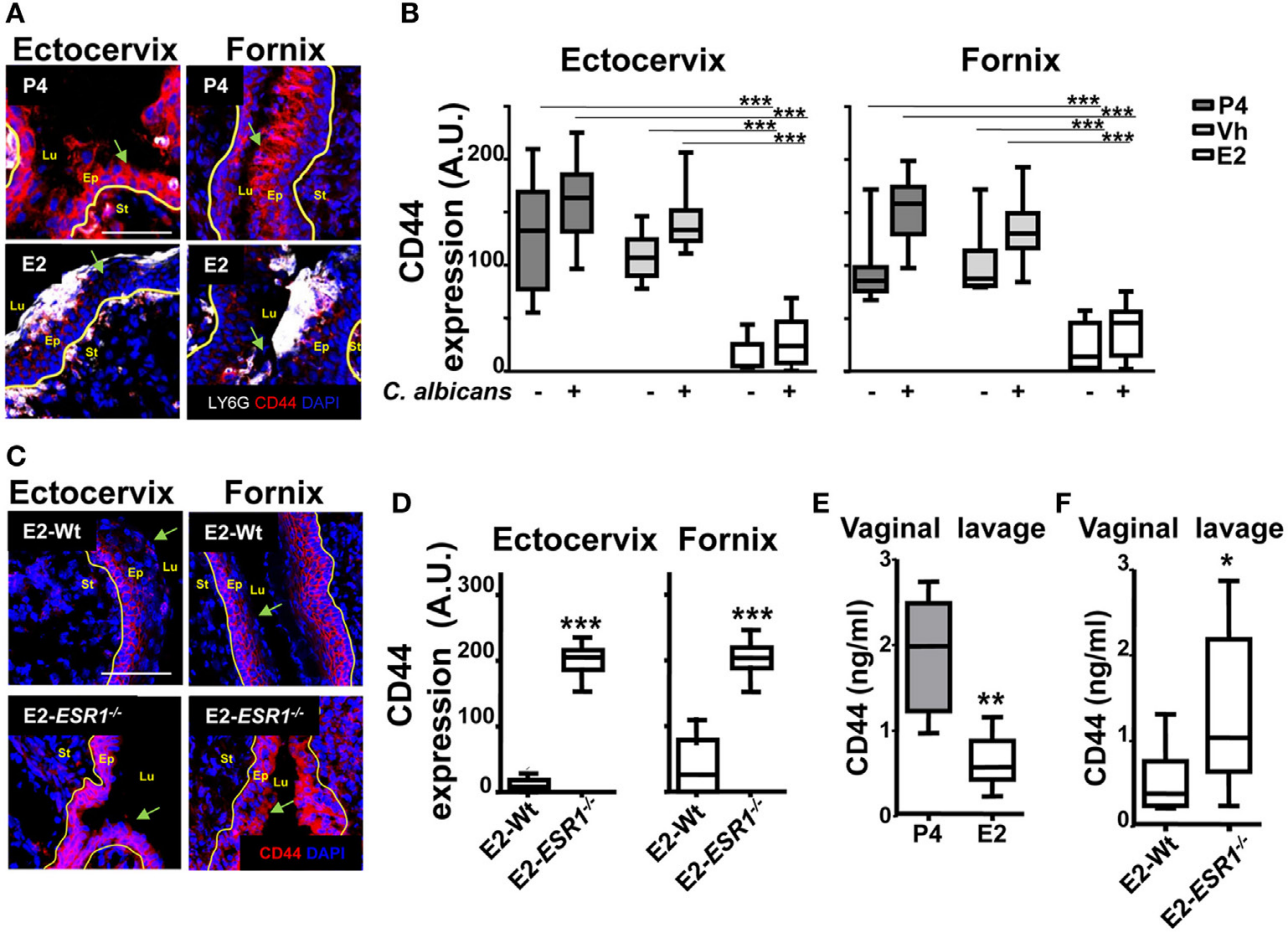
CD44 expression in the female reproductive tract (FRT). Hormone-treated ovariectomized mice were mock or *Candida albicans* inoculated in the vagina. **(A)** FRT indicated anatomical regions CD44-stained (red) and LY6G-stained neutrophils (white). **(B)** CD44 expression in the apical epithelium. Data were calculated in at least three different points in three different sections of each sample (*n* = 5). **(C)** Estradiol treated *ESR1*^−^*^/^*^−^ and Wt mice challenged with *C. albicans*. Representative photographs of ectocervix and fornix stained with CD47 (red) and **(D)** quantification (*n* = 5). **(E)** CD44 was assayed by ELISA in the vaginal lavage of hormone treated *C. albicans* inoculated mice and **(F)** estradiol-treated *ESR1*^−^*^/^*^−^ and Wt mice challenged with *C. albicans*. Data are expressed as box and whiskers 10–90 percentile (*n* = 5). *p < 0.05, **p < 0.01, and ***p < 0.001 Mann–Whitney. Yellow line indicates the lamina propia. Scale bar, 200 µm. E2, estradiol; Vh, vehicle; P4, progesterone. Abbreviations: Lu, lumen; Ep, epithelium; St, stroma; A.U., arbitrary units.

Finally, after migrating across epithelial cells, neutrophils emerge in the apical epithelium, where they need to detach from the epithelial cells before they reach the lumen (9, 10). CD44v6 and CD55 are membrane glycoproteins expressed on the apical surface of gut epithelial cells that play an anti-adhesive role and promote neutrophil release to the lumen (27). We detected that E2-treated mice displayed retention of neutrophils after vaginal lavage, which remained attached to the supra-epithelium, facing the vaginal lumen (Figure 2B). Therefore, we explored CD44 expression in VK2 vaginal epithelial cells. We found apical epithelial expression of CD44 displaying the glycan sialyl Lewis A (sLe^a^) (Figure 7A). Importantly, sLe^a^ on CD44v6, recognized by the mAb GM35, has previously been shown to be required for neutrophil entry into the intestinal lumen (34). Furthermore, blockade with mAb GM35 resulted in a reduction (~90%) in PMN TEM across VK2 cells (Figure 7B). Thus, CD44v6/sLe^a^ is necessary for the release of neutrophils from the apical surface of the vaginal epithelial cells in migration assays. Similarly, we detected higher surface expression of CD44 in *ex vivo* FRT epithelial cells isolated from P4- compared to E2-treated mice (Figure 7C). Next, we explored the anatomical localization of the CD44 in the mouse lower FRT. We detected increased expression of epithelial CD44 in P4- and Vh-treated compared with E2-treated mice in the vagina (~9-fold), ectocervix (~9-fold), and fornix (~6-fold). In addition, CD44 expression was unaltered by candida infection (Figures 8A,B; Figure S3B in Supplementary Material), indicating, a candida independent and E2-dependent effect. Indeed, E2-teated *ESR1*^−/−^ mice showed strong CD44 expression in the ectocervix and fornix, indicating the key role of E2 in the CD44 repression (Figures 8C,D). Notably, E2 treatment strongly reduced the levels of soluble CD44 present in the vaginal lavage and in the surface of epithelial cells from the mouse lower FRT. This effect was abolished in the E2-teated *ESR1*^−/−^ mice (Figures 8E,F). In contrast to CD44, expression of CD55 was unaffected by the hormones (Figure S3C in Supplementary Material). Hence, our data are in agreement with a role of CD44 in neutrophil TEM previously reported in the gut, in which shedding of the extracellular domain of CD44v6 is associated with the release of neutrophils from the apical epithelial surface (9, 34). In summary, E2 downregulates CD44 expression and shedding in the apical fornix and ectocervix epithelial cells, which together with the role of this receptor in TEM suggests that estradiol impairs neutrophil detachment, thus reducing their entry into the vaginal lumen during ovulation.

### Effect of Female Sex Hormones on CD44 and CD47 Expression in Gut and Bladder Epithelial Cells

**Figure 9:**
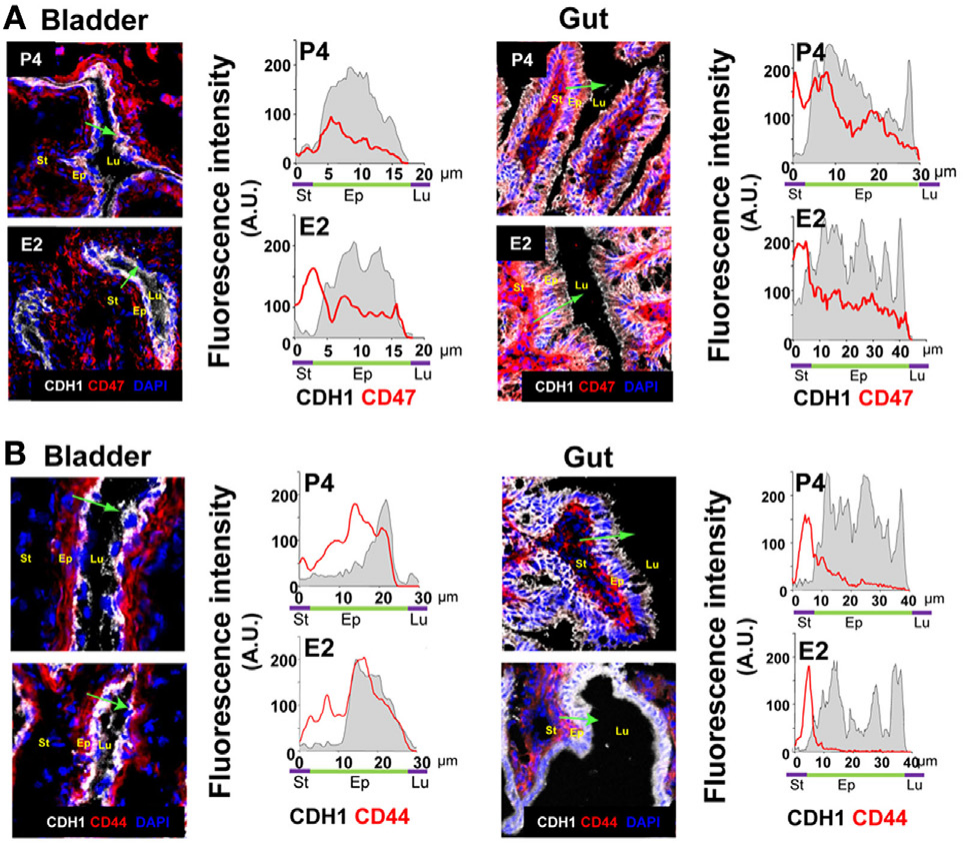
Bilaterally ovariectomized mice received hormone treatment subcutaneously (*n* = 5). **(A)** Bladder and gut CD47-stained. CD47 expression in the epithelium (CDH1+) cross section. **(B)** Bladder and gut CD44-stained. CD44 expression in the epithelium (CDH1+) cross section. Green arrow shows the cross section direction. Scale bar, 200 µm. E2, estradiol; P4, progesterone. Abbreviation: A.U., arbitrary units; Lu: lumen; Ep: Epithelium; St: Stroma.

Because hormonal treatment has a systemic effect, we wondered if E2-treatment also lowered epithelial expression of CD47 and CD44 in other mucosal lined organs. We observed a similar constitutive expression of CD47 in both gut and bladder epithelial cells. However, although constitutive epithelial expression of CD44 was observed in the bladder, CD44v6 expression in the gut was only observed following an inflammatory stimulus (34, 35). Significantly, E2 treatment had no effect on expression of CD44 and CD47 in gut and bladder epithelia (Figures 9A,B). Therefore, E2 specifically modulates expression of receptors required for TEM in the lower FRT epithelium.

### Role of Neutrophils Attached to the Apical Fornix and Ectocervix Epithelium

**Figure 10:**
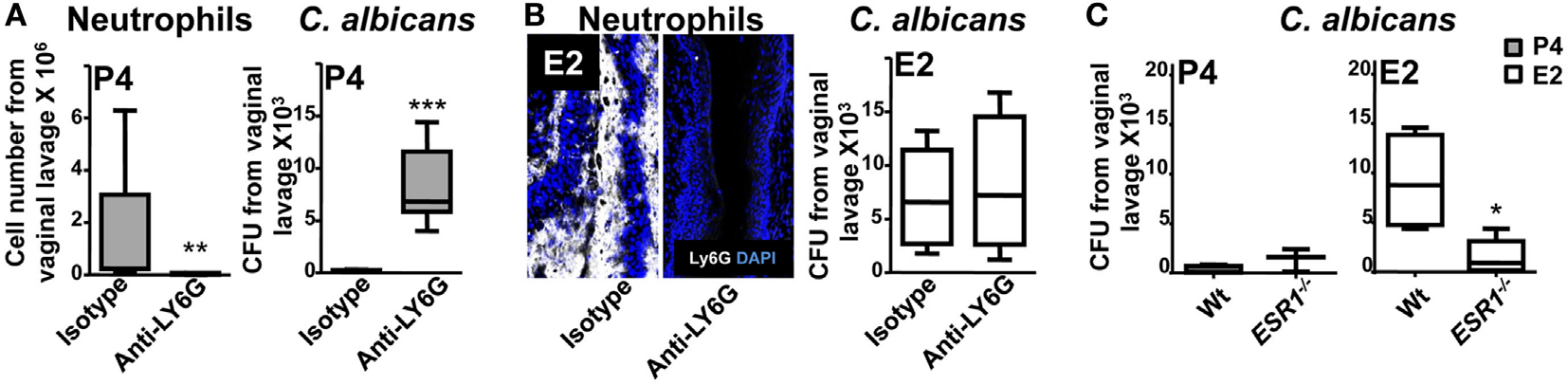
Hormone regulation of neutrophil killing activity. Bilaterally ovariectomized mice received hormone-treatment subcutaneously and rat anti-mouse LY6G (1A8) or isotype control antibody intravenously 24 h prior to *Candida albicans* inoculation to deplete the neutrophils. **(A)** Progesterone and **(B)** estradiol treated mice 6 h after the infection. Number of neutrophils and fungal burden (CFU) in the vaginal lavage after the infection (*n* = 6–8). **(C)** Fungal burden (CFU) in the vaginal lavage of progesterone or estradiol-treated *ESR1^−/−^* and Wt mice challenged with *C. albicans*. Data are expressed as box and whiskers 10–90 percentile (*n* = 5). *p < 0.05, **p < 0.01, and ***p < 0.001 Mann–Whitney. Abbreviations: CFU, colony forming unit. E2, estradiol; P4, progesterone.

We found that E2 reduces the expression on the epithelial cells of key receptors involved in TEM and retains neutrophils attached at the fornix and ectocervix epithelium. To evaluate the neutrophil potential killing activity, we challenged them in the *in vivo C. albicans*-infected model. P4-treated neutrophils showed a robust candidacidal activity (~85% reduction) *in vivo*, and depletion of neutrophils impaired the vaginal fungal clearance (Figure 10A). However, E2-treated mice were inefficient clearing the fungal infection when they were attached to the epithelium (Figure 10B). Further, fungal burden was reduced (~6-fold) in *ESR1*^−/−^ mice suggesting that ESR1 is necessary for the E2-dependent impaired candidacidal activity; whereas it had no effect on the P4-treated Wt and *ESR1*^−/−^ mice (Figure 10C). Therefore, P4-treatment induces neutrophil dependent protection, and E2-treatment reduces neutrophil killing potential during ovulation, probably because of E2 dependent vaginal epithelial cells secretions (36) and induces susceptibility to vaginal infection, which is ESR1 dependent. Thus, we suggest that neutrophil retention could avoid sperm damage during ovulation to favor reproduction, although it induces susceptibility to *C. albicans*.

## Discussion

**Figure 11:**
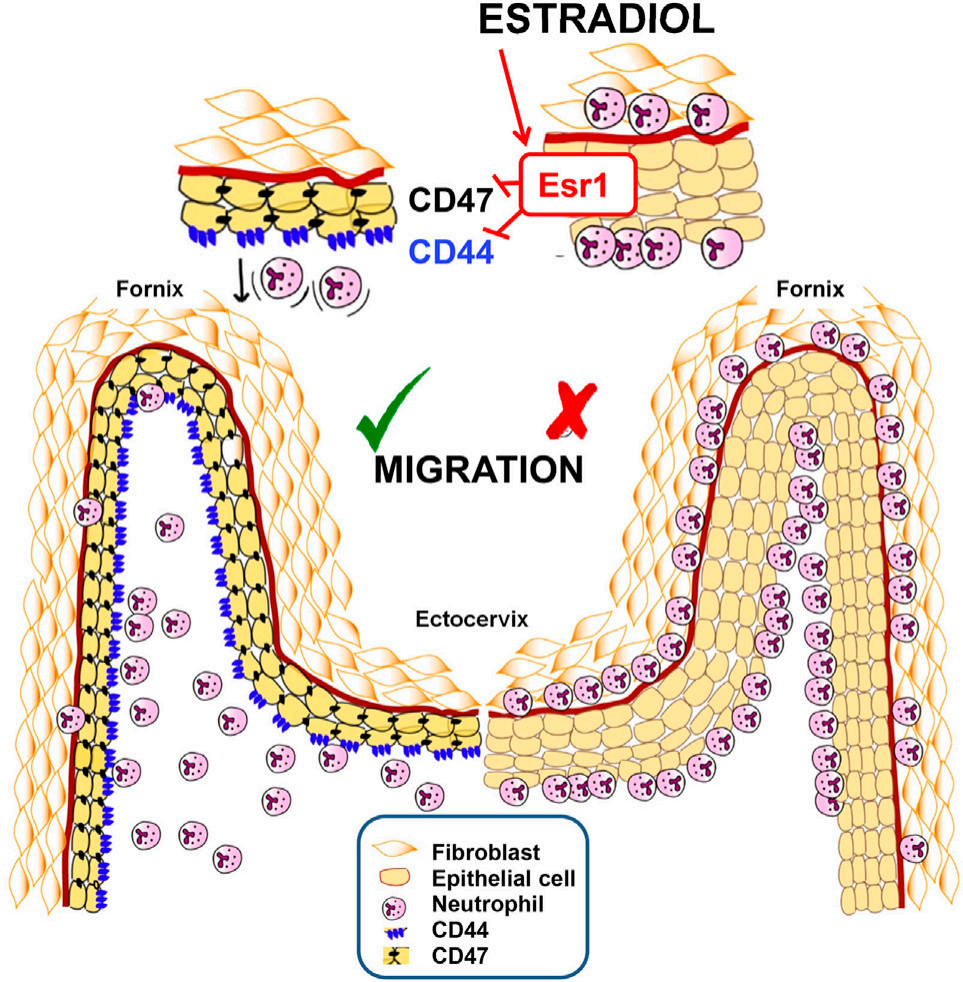
Summarizing schematic figure. Progesterone treatment promotes neutrophil migration to the vaginal lumen. In contrast, estradiol treatment downregulates CD44 and CD47 expression in the cervix and fornix epithelial cells, by an ESR1-dependent mechanism, which accumulates neutrophils in the sub- and supra-epithelial spaces of the ectocervix and vaginal fornix rather than at the vaginal lumen.

We present an ESR1-dependent mechanism that significantly decreases neutrophil protection in the vaginal lumen during ovulation or E2-based therapies. E2 treatment alters CD44 and CD47 expression in the FRT epithelium, which accumulate neutrophils in the sub- and supra-epithelial spaces of the ectocervix and vaginal fornix rather than at the vaginal lumen. In contrast, P4-treatment promotes neutrophil migration to the vaginal lumen and neutrophil killing (Figure 11). Unique to vaginal mucosa, neutrophil TEM is independent of the infection but dependent on sex hormones to prevent sperm from neutrophil attack, although it may compromise immunity during ovulation. These data help to explain why E2-based hormonal replacement therapies predispose women to vaginal infections and provide a new mechanism to explain the long-standing observation that vaginal neutrophil levels vary during the ovarian cycle (16).

Neutrophils protect the lower FRT from opportunistic microbiota/sexual transmitted diseases-causing pathogens. During the luteal phase, neutrophils continuously enter the vaginal lumen through the ectocervix (17). However, during the ovulatory phase (high E2 levels), neutrophils disappear from the vaginal lumen (16), probably because E2-treatment alters CXCL1 gradients (23) and CD47 expression in the lower FRT epithelial cells, which might retard neutrophil migration. In addition, E2-treatment downregulates apical expression of CD44v6, thereby impairing neutrophil detachment. Besides, neutrophils have abolished their killing potential, probably because of E2-dependent vaginal epithelial cells secretions like heparan sulfate, which could affect the neutrophils interaction with candida (36).

Here, we present evidences of a mechanism by which E2 treatment acting on the epithelium, but not on the neutrophils, accumulates them at the ectocervix and fornix, where they provide barrier protection, and could contribute to tissue repair (37). Neutrophils accumulated in these areas would be prepared to quickly invade the vaginal lumen during the early luteal phase (17), when P4 restores neutrophil infiltration (23) by favoring neutrophil detachment and release to the vaginal lumen. Moreover, P4 reestablishes TEM of the sub-epithelial neutrophils to promote a second wave of immune cells that control the vaginal microbiota and trap sperm or sperm-associated microbes after ovulation (38). We propose that this could be an ESR1-driven, multilayer safety mechanism to prevent unspecific neutrophil damage.

Epithelial cells represent the first barrier of defense against pathogens and produce signals in response to aggressions that attract the innate immune cells from the blood. Several tissues such as the gut, bladder, and lungs, constitutively present CD47 expression (8, 30, 39). In contrast, tissue-specific epithelial expression of CD44v6 has been reported (34). In any case, inflammatory signals upregulate epithelial expression of CD47 (40) and CD44v6 (34) to favor neutrophil TEM. However, unique to the lower FRT mucosa, epithelial CD44 and CD47 expression was independent on the candida infection, because neutrophil infiltration and functions must be timed to the ovarian cycle. Our data support the notion that E2 acts on several steps of neutrophil TEM independently of infection with *C. albicans* to anticipate immune protection, while protecting sperm from neutrophil attack, even at the risk of a compromised immunity during ovulation.

The absence of neutrophils in the vaginal lumen during ovulation was first reported in 1917 (16), but its mechanisms have remained unknown. We present a unique model by which sex hormones regulate neutrophil influx to the vaginal lumen, which is in a coordinated fashion that allows for both containment of infections and reproductive functions. Our data reveal that ESR1 is necessary for the E2-dependent downregulation of neutrophil TEM and impairs neutrophil killing functions during ovulation to protect sperm. Clinically, these data imply that E2-based treatments could compromise vaginal immunity and favor opportunistic microbiota or STDs causing pathogen infections.

## Ethics Statement

Procedures involving human and animals samples complied with national and international laws and policies. The Ethical Board of the IiSGM approved the human samples collection (CEIC-JV1.1/07-2014) and animal procedures (PROEX14714 and 27615).

## Author Contributions

MR, LS, and RS conceived the study. LS, RC, EM, IO, CF-P, LM, JG, RS, and MR carried out the experiments. FA, MM, AH, JB, and CP provided key reagents, help, and protocols. LS, RS, and MR analyzed the data. MR wrote the manuscript and LS, PS, JB, AH, CP, and RS revised the manuscript. All the authors approved the submitted version.

## Conflict of Interest Statement

The authors declare that the research was conducted in the absence of any commercial or financial relationships that could be construed as a potential conflict of interest.

## Supplementary Material

Figure S1 Representative flow cytometry plots of PMN purification analysis by flow citometry. Representative dot plots of cells stained with CD16 and CD3 to assay purity and 7AAD as a marker of cell death. A representative experiment of three independent tests is shown.

Figure S2 Hormone-treated ovariectomized mice were mock or *Candida albicans* inoculated in the vagina. **(A)** Female reproductive tract (FRT) indicated anatomical region sections were LY6G-stained neutrophils (white). **(B)** LY6G+ cells/field in the indicated areas of the FRT epithelium and stroma. Data were calculated in at least three different sections of endocervix or uterus or vagina (*n* = 5) and are expressed as box and whiskers 10–90 percentile (*n* = 8 to 10 mice per time points). ns, no significant; **p* < 0.05, ***p* < 0.01, ****p* < 0.001, Mann–Whitney. Scale bar, 200 µm. Yellow line indicates the lamina propia. Abbreviations: E2, estradiol; P4, progesterone; Lu, lumen; Ep, epithelium; St, stroma.

Figure S3 Hormone treated ovariectomized mice were mock *Candida albicans* inoculated in the vagina. **(A)** Vaginal epithelium stained for CD47 (red) or neutrophils (white). CD47 expression in the basolateral epithelium. Data were calculated in at least three different points in three different sections of each sample (*n* = 5). **(B)** Vaginal epithelium CD44-stained (red) and LY6G-stained neutrophils (white). CD44 expression in the apical epithelium. Data were calculated in at least three different points in three different sections of each sample (*n* = 5). Data are expressed as box and whiskers 10–90 percentile. ****p* < 0.001 Mann–Whitney. **(C)** Female reproductive tract indicated anatomical regions CD55 stained (*n* = 3). Scale bar, 200 µm. Yellow line indicates the lamina propia. Abbreviations: E2, estradiol; P4, progesterone. Lu, lumen; Ep, epithelium; St, stroma.
