# Supplementary figures and images for: Estrogen Receptor-Alpha (ESR1) Governs the Lower Female Reproductive Tract Vulnerability to Candida albicans
Source: Front Immunol. 2018 May 24;9:1033. doi: 10.3389/fimmu.2018.01033 (PMC5976782; doi:10.3389/fimmu.2018.01033)

Supp Figure 1

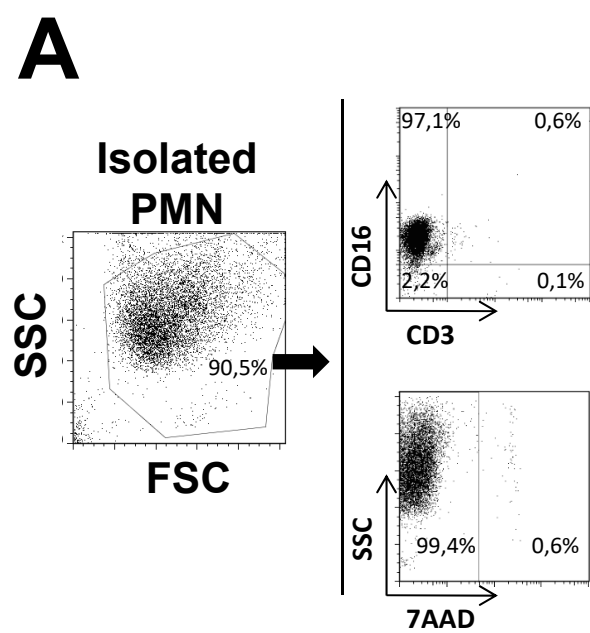

Supp Figure 2

**A**

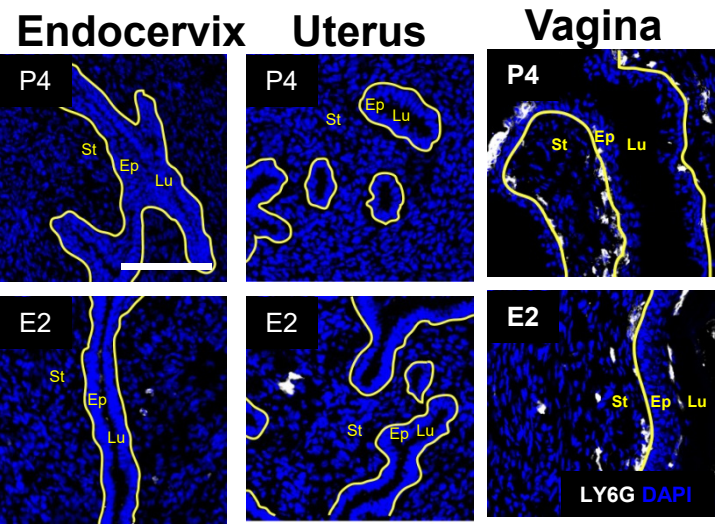

**B**

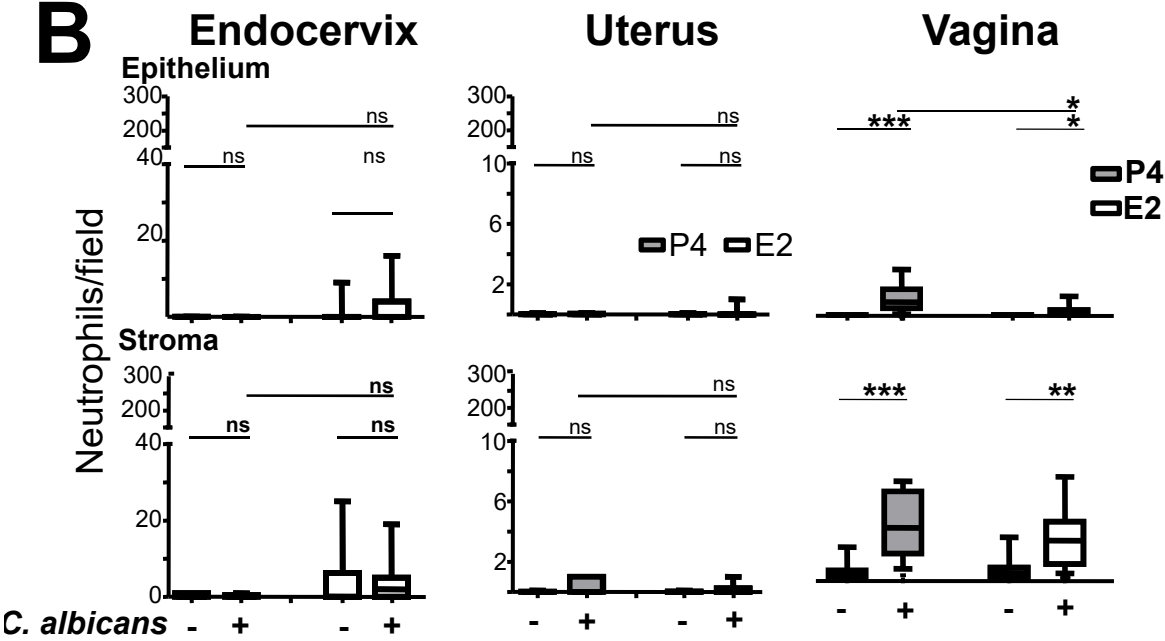

Supp Figure 3

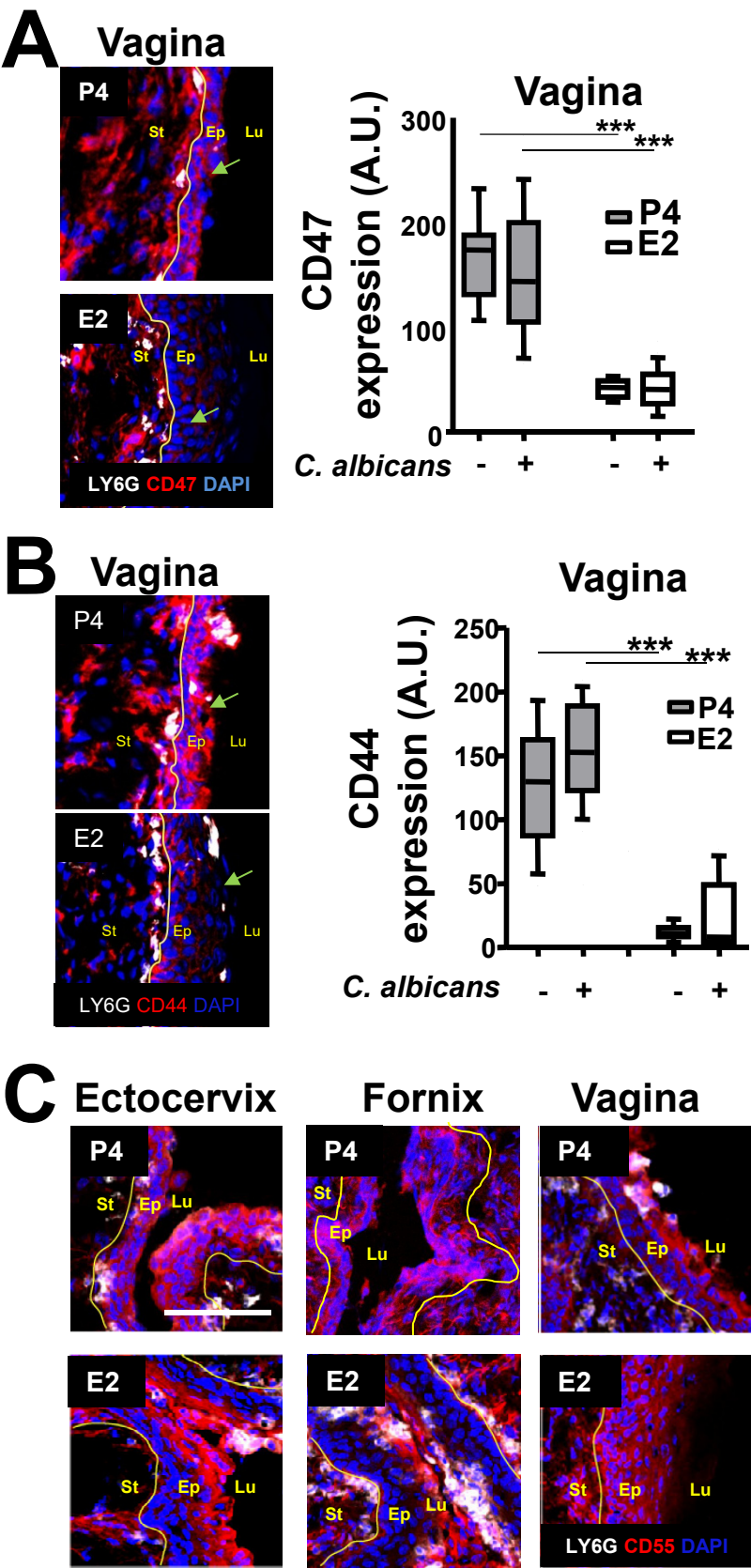

Supplement: Figure S1 — Representative flow cytometry plots of PMN purification analysis by flow citometry. Representative dot plots of cells stained with CD16 and CD3 to assay purity and 7AAD as a marker of cell death. A representative experiment of three independent tests is shown. [file presentation_1.PDF]
